# Functions of ADP-ribose transferases in the maintenance of telomere integrity

**DOI:** 10.1007/s00018-022-04235-z

**Published:** 2022-03-29

**Authors:** Daniela Muoio, Natalie Laspata, Elise Fouquerel

**Affiliations:** 1 UPMC Cancer Institute and Department of Pharmacology and Chemical Biology at the University of Pittsburgh, Hillman Cancer Center, 5115 Centre Avenue, Pittsburgh, PA 15213 USA; 2 Department of Biochemistry and Molecular Biology, Thomas Jefferson University, 233 S. 10th street, Philadelphia, PA 19107 USA

**Keywords:** ART, PARP, Telomeres, Genome stability

## Abstract

The ADP-ribose transferase (ART) family comprises 17 enzymes that catalyze mono- or poly-ADP-ribosylation, a post-translational modification of proteins. Present in all subcellular compartments, ARTs are implicated in a growing number of biological processes including DNA repair, replication, transcription regulation, intra- and extra-cellular signaling, viral infection and cell death. Five members of the family, PARP1, PARP2, PARP3, tankyrase 1 and tankyrase 2 are mainly described for their crucial functions in the maintenance of genome stability. It is well established that the most describedrole of PARP1, 2 and 3 is the repair of DNA lesions while tankyrases 1 and 2 are crucial for maintaining the integrity of telomeres. Telomeres, nucleoprotein complexes located at the ends of eukaryotic chromosomes, utilize their unique structure and associated set of proteins to orchestrate the mechanisms necessary for their own protection and replication. While the functions of tankyrases 1 and 2 at telomeres are well known, several studies have also brought PARP1, 2 and 3 to the forefront of telomere protection. The singular quality of the telomeric environment has highlighted protein interactions and molecular pathways distinct from those described throughout the genome. The aim of this review is to provide an overview of the current knowledge on the multiple roles of PARP1, PARP2, PARP3, tankyrase 1 and tankyrase 2 in the maintenance and preservation of telomere integrity.

## Introduction

**Fig. 1:**
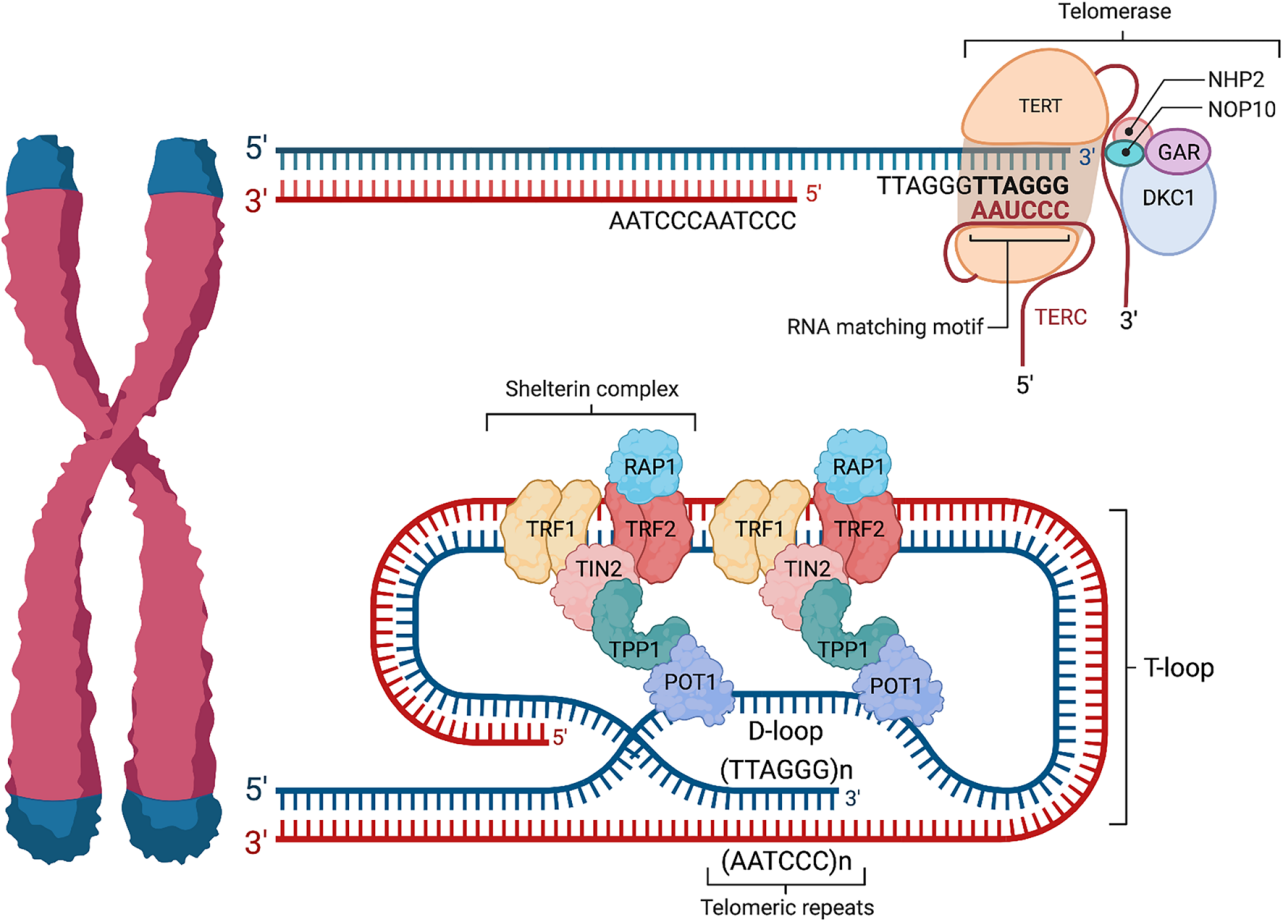
Telomere structure, shelterin complex and telomerase. Telomeres are composed of long arrays of tandem TTAGGG repeats that are heterogenous in length and can reach up to 15 kb. The G-rich lagging strand ends in a 3′ single-stranded overhang of about 50–200 nucleotides. The overhang folds back and pairs with the C strand in the double-stranded DNA, which form a D-loop that results in a higher-order DNA structure called the t-loop. The TTAGGG/CCCTAA duplexes are bound by TRF1 and TRF2, two members of the shelterin complex. The subunit TIN2 (TRF1-interacting nuclear factor 2) bridges TRF1 and TRF2 while simultaneously interacting with a third partner, the subunit TPP1 (adrenocortical dysplasia protein homolog, also known as ACD). TPP1 interacts with POT1 (protection of telomere 1), which binds the 3′ single-stranded overhang via its two OB fold domains and consolidates the nucleoprotein complex cohesion. Finally, RAP1 (TRF2-interacting protein), the Shelterin’s last subunit, binds exclusively to TRF2. Telomerase is a retro-transcriptase whose holoenzyme is composed of a telomerase reverse transcriptase (hTERT) and an RNA component (hTERC or hTR) as well as several other protein factors, such as dyskerin (DKC1), NHP2, NOP10, and GAR1, which are also part of the H/ACA ribonucleoprotein complex. hTERC contains a sequence complementary to the telomeric DNA and serves as a matrix for telomere elongation

Telomeres are specialized nucleoprotein structures located at the ends of linear chromosomes that are crucial for chromosome replication and maintenance. Telomeres protect chromosome ends from erosion and subsequent loss of genetic material, thereby preventing genomic instability that can cause numerous human diseases. Human telomeric DNA consists of arrays of tandem hexanucleotide TTAGGG repeats that vary in length and can reach up to 15 kb. The G-rich lagging strand ends in a 3′ single-stranded overhang of about 50–200 nucleotides that folds backward and pairs with the C strand of  the upstream double-stranded DNA (dsDNA). This association locally displaces the G strand, forming a D-loop that further results in a higher-order chromatin structure called a t-loop. This t-loop protects the chromosome ends from being wrongfully identified as dsDNA breaks (DSBs) that can lead to end-to-end chromosome fusions, thereby bypassing the end-protection problem and preventing genome instability [1–3] (Fig. 1). Another prominent feature of telomeres is the association of a six-subunit protein complex called Shelterin that is not located anywhere else on the genomic DNA and is present throughout the cell cycle. TRF1 and TRF2 (telomeric repeat-binding factor 1 and 2) bind with high affinity to the duplex telomeric DNA as homodimers via their respective Myb domain [4–7]. The homodimerization of both TRF1 and TRF2 is mediated by their TRFH (Telomeric Repeat Factors Homology) domain. Interestingly, when bound to dsDNA, the TRF2 dimer is able to promote the wrapping of 90 bp of telomeric DNA via interaction with its TRFH domains, which exerts a topological stress that is thought to aid T-loop formation [8]. Other proteins in the complex include TIN2, TPP1, POT1 and RAP1 (Fig. 1). The Shelterin complex counteracts the end-protection problem by preventing the action of at least 7 DNA damage response (DDR) pathways that could trigger gross genomic instability if activated in an unsolicited manner (reviewed in [7]) [9] (Fig. 1). Another key telomere-specific factor is the enzyme telomerase. Telomerase is a ribonucleoprotein composed of a telomerase reverse transcriptase TERT, which carries its own RNA template called TR (also referred to as TERC). Telomerase maintains telomere length by adding TTAGGG repeats using TR as a template, effectively solving the end-replication problem [10–12]. Several other accessory factors and DNA repair proteins also function in the protection of telomeres (reviewed in [13]) (Fig. 1). In this review, we will particularly focus on the roles played by  the five ARTs PARP1, PARP2, PARP3, tankyrase 1 and tankyrase 2. We will provide an overview of the roles played by these enzymes in both the maintenance of telomere homeostasis and telomeric DNA repair upon genotoxic insult. We also highlight controversies and discuss the potential open questions that remain to be addressed.

## The ART enzyme family and the ADP-ribosylation reaction

The ART family consists of 17 enzymes responsible for the transfer of ADP-ribose from NAD^+^ onto protein target amino acids, a reversible post-translational modification of proteins called ADP-ribosylation. ARTs are involved in numerous cellular processes including DNA damage response, protein and lipid metabolism, immune response, chromatin remodeling, transcription regulation and cell death [14–16]. All family members share an ART diphtheria-like (ARTD) domain responsible for mono- or poly-ADP-ribosylation (MARylation or PARylation) [17]. Among the 17 members of the family, only PARP1, PARP2, tankyrase1 and tankyrase2 synthesize polymers of ADP-ribose units, leading to the formation of PAR chains. PARP1 and PARP2 can form up to 200 ADP-ribose units-long and branched PAR while tankyrase 1 and tankyrase 2 synthesize shorter, unbranched PAR of about 20 units (oligo-PAR) [17, 18]. Other family members such as PARP3 are MAR transferases (also PARP6-12 and PARP14-16) and do not possess any catalytic activity (PARP13). Both MAR and PAR modifications can also be recognized by protein readers via specific domains or motifs. The first characterized module is the PAR binding motif, or PBM, that is structurally undefined and present in over 800 proteins. The PBM mediates PAR binding through electrostatic interactions between its positively charged amino acids and the negatively charged PAR [19]. Other protein interactions involve specialized domains like the macrodomain that binds both MAR and PAR, the BRCT domain or the domains involved in nucleic acid binding, such as the OB fold domain or the RNA recognition motif (RRM) [20]. Covalent and non-covalent binding of proteins to MAR and PAR allow for the regulation of their activities and the modulation of their interactions with other proteins or substrates. The transient nature of MARylation and PARylation is ensured by the activities of specialized ADP-ribose hydrolases. One of the first characterized enzymes for the removal of PAR chains is the poly(ADP-ribose) glycohydrolase (PARG) [21], which harbors both *exo*- and *endo*-glycohydrolase activities and binds to PAR through a macrodomain [22]. Another enzyme found to degrade PAR chains is the ADP-ribosylhydrolase 3 (ARH3) which has a different structure than PARG and a slower catalytic activity [23, 24]. However, PARG is unable to cleave the ADP-ribose unit that is directly bound to the targeted amino acid [22, 25]. The removal of this terminal PAR or MAR unit is ensured by other macro-domain containing enzymes including macroD1, macroD2 and terminal ADP-ribose glycohydrolase (TARG) as well as ARH1 [26–28]. PARylation affects DNA–protein binding through different mechanisms: upon being PARylated, some proteins exhibit decreased affinity for DNA while others exhibit increased affinity. This modulation is essential for various cellular processes, and the balance between synthesis and degradation is therefore crucial to avoid the toxic accumulation of PAR in cells, which could lead to cell death [29].

### DNA-dependent ARTs PARP1, PARP2 and PARP3

**Fig. 2:**
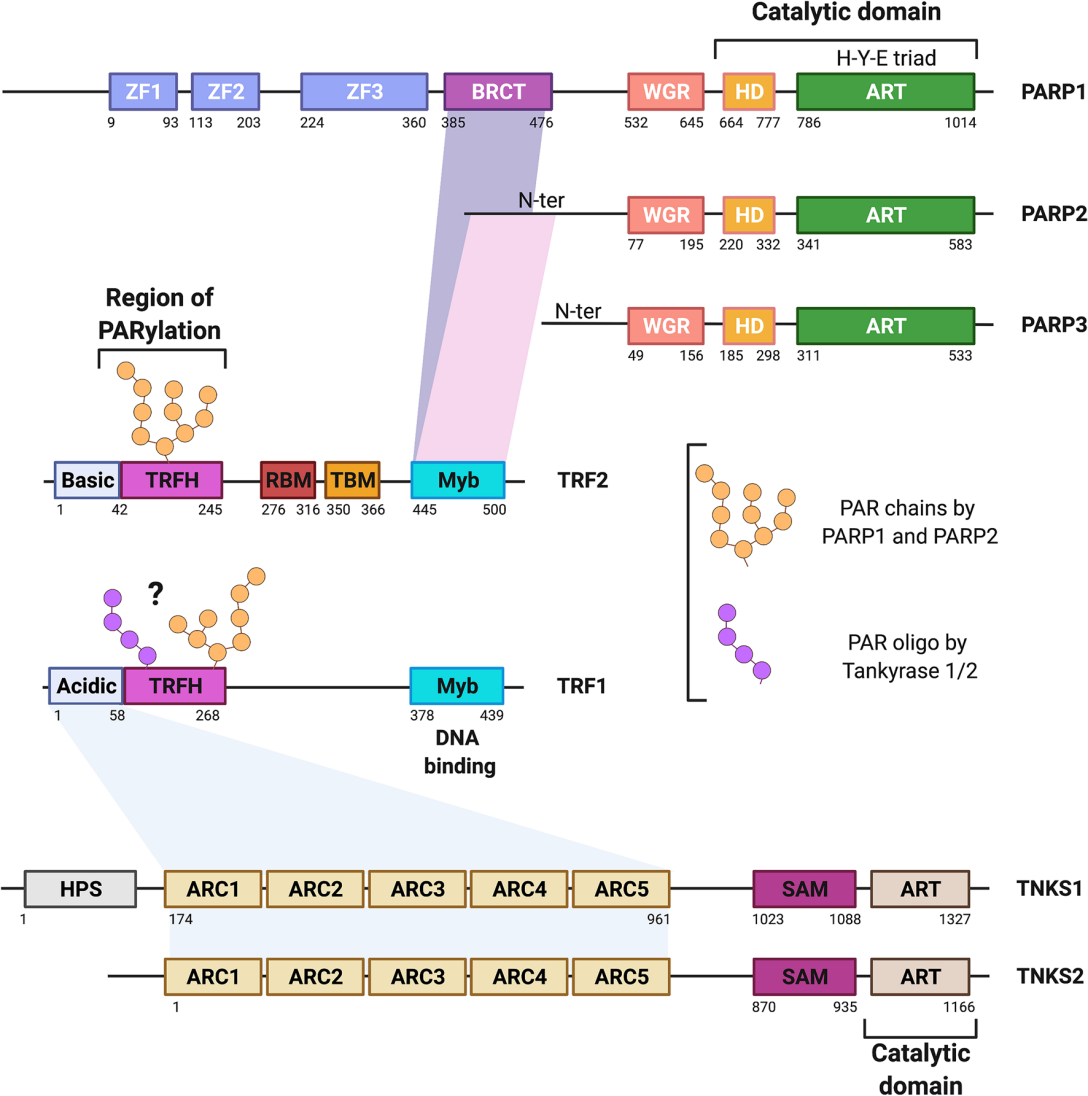
Modular structures of the ART enzymes PARP1, PARP2, PARP3, tankyrase 1, tankyrase 2 and the shelterin proteins TRF1 and TRF2. The amino acid numbers indicate domain boundaries. The known interactions between the ART enzymes and shelterin proteins and the domains involved are indicated by purple (PARP1/TRF2), pink (PARP2/TRF2) and light blue (Tankyrases1/2/TRF1) shadings. The PARylated domains of TRF1 and TRF2 are also indicated (see text for more details)

PARP1, PARP2 and PARP3 are DNA-dependent ARTs whose activity is triggered by DNA breaks. They are therefore involved in several DNA repair mechanisms including base excision repair (BER) and both the single- and double-strand break repair pathways (SSBR and DSBR). The modular structure of PARP1 consists of a DNA-binding domain that contains three zinc fingers (Zn1, Zn2, and Zn3). It is followed by a BRCT domain that mediates protein–protein interactions and is a target of auto-modification, a WGR domain containing Trp–Gly–Arg residues, and a C-terminal catalytic domain comprising an α-helical domain (α-HD) associated with the ART domain [30–32]. The three Zn fingers and WGR domain are responsible for DNA binding [33]. However, PARP2 and PARP3 contain only a WGR domain, explaining differences in substrate specificity (Fig. 2). PARP2 and PARP3 are mostly activated by DNA nicks, breaks and gaps that possess 5′ phosphorylated ends [34]. PARP1 however has a much broader substrate specificity and can bind and be activated by single- and double-strand breaks (SSBs and DSBs) regardless of their end termini, DNA crosslinks, stalled replication forks, DNA hairpins, cruciform structures, as well as G quadruplexes (G4s) [34–36]. While PARP3 has been involved in the repair of DSBs via classical non-homologous end joining (c-NHEJ), PARP1 and PARP2’s most described roles are in the repair of SSBs via BER and SSBR. As the ART enzyme responsible for almost 90% of PARylation activity upon DNA damage induction, PARP1 is also central to DSB repair through roles in alternative end-joining (alt-EJ) and homologous recombination (HR) [37]. Upon activation, the PAR synthesized by PARP1 and PARP2 serves as a docking platform for the recruitment of DNA repair proteins or enzymes involved in the resolution of the damage or the harmful DNA structures. During BER, PARP1 and PARP2 recognize and bind to the SSB intermediate generated by the removal of the damaged base through the combined activities of a specialized glycosylase and the endonuclease APE1 [38]. The primary targets of PARylation are PARP1 and PARP2 themselves. They both interact with downstream DNA repair proteins XRCC1, Ligase III and polymerase beta (Polβ) via protein–protein interactions or PAR binding [39–41]. Another important function of PARylation is the hetero-modification of histones surrounding the lesion, which triggers a local decondensation of the chromatin, thereby easesing the recruitment of DNA repair proteins [16]. Interestingly, it was recently reported that DNA damage specifically triggers PARylation of serine residues on protein targets rather than the known aspartates, glutamates, arginines or lysines [42]. PARylation on serines is promoted by histone PARylation factor 1 (HPF1) that binds to PARP1 and PARP2 to complete their catalytic site and to enable the nucleophilic attack on the serine residue specifically [43, 44]. In this context, the removal of the last ADP-ribose unit on the serine residue is ensured specifically by ARH3 [43]. By degrading PAR, PARG and ARH3 ensure a rapid turnover of PARP1 and PARP2 molecules and contributes to the efficiency of the repair [45].

**Fig. 3:**
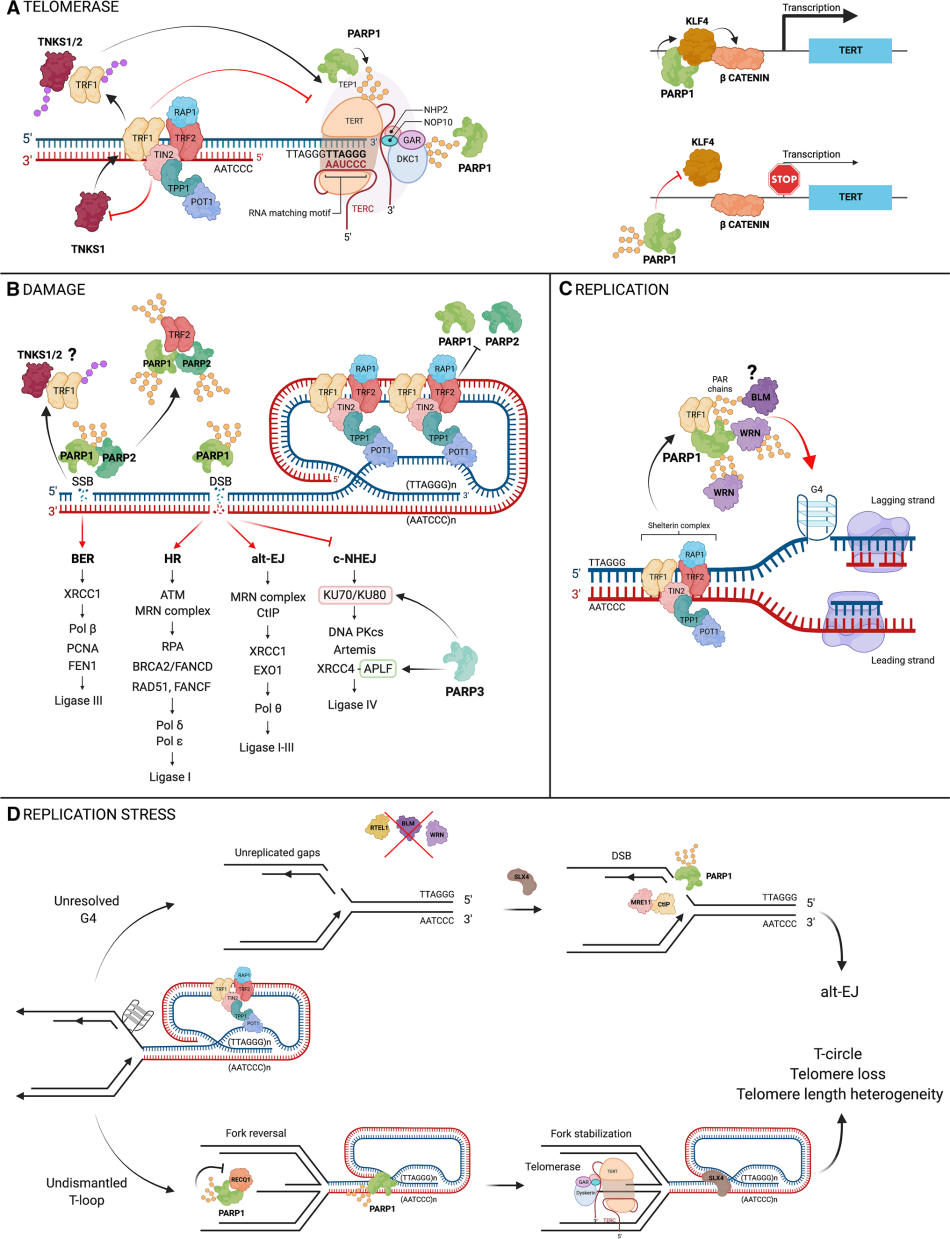
Schematics of the different telomere maintenance pathways involving ART enzymes. **A** Tankyrase-dependent PARylation of TRF1 displaces the protein from the DNA, which allows telomerase access to telomeres. TIN2 negatively regulates tankyrase 1. PARP1 can target protein components of the holoenzyme and/or control hTERT gene expression through PARP activity-independent regulation of KLF4. **B** Tankyrase activity is activated upon oxidative stress at telomeres and PARylates TRF1. PARP1 and PARP2 are recruited at telomeres upon oxidative stress and can PARylate TRF2 in vitro. Internal telomeric DSBs are repaired via PARP1-dependent alt-EJ. **C** During replication, telomeric G4s are unwound by the RECQ helicases WRN and BLM. PARP1 is activated upon treatment of cells by G4 ligands, which trigger the PARylation of TRF1 and recruitment of WRN and BLM. PARP1 is also able to PARylate WRN directly while WRN binds PAR in vitro. **D** Telomeres are highly sensitive to replication stress due to their t-loop structure and the formation of G4s. Preventing G4 unwinding by BLM leads to DSBs, which are repaired by PARP1-dependent alt-EJ. Deficiency in helicase RTEL1, as observed in several telomeropathies, leads to telomeric dysfunction due to an un-resolved t-loop. Stalling of the replication fork triggers fork reversal, whose chicken foot structure is recognized by telomerase. PARP1 prevents fork restart by PARylating RECQL1, which leads to telomeric dysfunction

Upon DSB induction, cells can choose between HR, c-NHEJ and alt-EJ pathways for the repair. Although NHEJ is active throughout the cell cycle, HR, which utilizes the sister chromatids as a template for repair, operates only during S and G2 phases [46, 47]. C-NHEJ involves the direct ligation of the two DSB ends and is promoted by the binding of the heterodimer Ku70/Ku80, which protects the ends from degradation [48]. This step is followed by the recruitment of DNA-PK kinase and subsequent ligation by XRCC4/Ligase IV [49]. During c-NHEJ, PARP3 accelerates DSB repair by recruiting Aprataxin-and-PNK-like factor (APLF), which promotes XRCC4-Ligase IV-mediated ligation [50] (Fig. 3B). In parallel, PARP3 interacts with and PARylates Ku70/Ku80, which was shown to help the recruitment and stabilization of the complex on the DNA ends [37, 51]. Despite these reports, the role of PARP3 in the repair mechanisms are less extensively studied and requires further investigation to be fully understood.

One key factor involved in HR is the complex comprising the endonuclease Mre11, the ATPase Rad50 and the protein NBS1 (MRN) [52–54]. MRN binds DNA ends to promote end resection in collaboration with CtIP [54, 55]. This step is followed by a more extensive resection catalyzed by exonuclease ExoI, which allows for the recruitment of the ssDNA protein RPA and the subsequent DNA coating by Rad51 that is required for strand invasion of the sister chromatid and the search of the template DNA [56, 57]. Interestingly, alt-EJ, which uses microhomology to repair the break, also relies on the MRN complex for minimal DNA strand resection [58] (Fig. 3B).

The roles of PARP1 in the HR pathway are two-fold: to prevent error-prone c-NHEJ by repressing Ku70/80 binding to the DSB ends and to promote the recruitment of the MRN complex through PAR binding to Mre11 via a PBM [59, 60]. Similarly, these PARP1-dependent mechanisms can also promote alt-EJ when c-NHEJ is compromised. Indeed, a role for PARP1 in the alt-EJ pathway was first suggested upon the observation that its depletion or inhibition impaired the DSB end rejoining in cells lacking Ku70/Ku80 or Ligase IV [61–63]. By binding to the DNA breaks instead of Ku70/Ku80, activated PARP1 is able to recruit XRCC1 and Ligase III (Fig. 3B).

### Tankyrases 1 and 2

Tankyrase 1 and tankyrase 2 are both TRF1-interacting ARTs and therefore fulfill telomere-specific tasks while also possessing distinct properties [64, 65]. They share 85% of sequence homology and thus have a comparable primary structure (Fig. 2). They both contain the ART domain but lack the HD domain present in the catalytic domain of PARP1, 2 and 3. They also contain a sterile alpha module (SAM), unique among the ART enzymes, that mediates their polymerization [66] and a cluster of five ankyrin repeats (ANK1-5). SAM mediates the polymerization of tankyrases while the ANK repeats facilitate the interaction of the enzymes with their protein targets, which possess a specific consensus sequence of 8 amino acids RxxxxG [67–70]. Tankyrase 2 differs from tankyrase 1 in that it lacks the N-terminal HPS domain (composed of homo-polymeric repeats of His, Pro and Ser residues), the function of which is still unknown [65] (Fig. 2). Tankyrase 1 and tankyrase 2 are not DNA-dependent enzymes; their activity is triggered upon binding to their protein targets. Both enzymes share the majority of their protein partners and consequently have overlapping biological functions, including in WNT/beta-catenin signaling, mitosis, apoptosis, viral replication and proteasome regulation [71]. However, the first identified functions of tankyrases are in the maintenance of telomeres via various mechanisms. These mechanisms are already extensively reviewed in a recent work [72]. Therefore, we will only briefly overview these mechanisms while highlighting a recently described role in the repair of damaged telomeric DNA.

## Tankyrases in the preservation of telomere integrity

### Tankyrases 1 and 2 in the regulation of telomerase

Tankyrase 1 was first discovered as a factor that regulates telomere length via the PARylation of TRF1. TRF1 is a major regulator of the telomerase enzyme as it controls its access to the telomeric DNA. Accordingly, overexpression of TRF1 leads to telomere shortening, while its depletion leads to telomere lengthening [73, 74]. TRF1 N-terminal region harbors a RxxxxG motif that makes it a protein partner of tankyrases 1 and 2, which are also able to PARylate it. PARylation of TRF1 prevents its binding to the telomeric DNA, thereby allowing the telomerase enzyme to access telomeres and elongate them [64, 75, 76] (Figs. 2 and 3A). In line with an overlapping role, the depletion of either of the tankyrases does not impact telomere length [77]. Another level of regulation is imposed by the shelterin protein TIN2 that binds both TRF1 and tankyrase 1 to prevent TRF1 PARylation [78]. However, the conditions in which this TIN2-dependent regulation is lifted to permit tankyrase activity-dependent telomere elongation have yet to be clarified (Fig. 3A).

### Tankyrases 1 and 2 in the resolution of telomere cohesion

Following replication and until the time of their separation into daughter cells during mitosis, sister chromatids are held together by a multi-protein complex called cohesin [79] that needs to be removed to guarantee proper segregation. Tankyrases 1 and 2 play key roles in this process by ensuring efficient telomere resolution in late S/G2 phases [80–82]. This was first demonstrated through the observation that the depletion of tankyrase 1 by siRNA prolonged anaphase and triggered an increase of sister chromatid telomere fusion, a sign of defective telomere resolution [80, 83]. Tankyrase-mediated telomere resolution was found to be regulated by the E3-ubiquitin ligase RNF8, which stabilizes tankyrase 1 in late S/G2 phases [81, 83, 84]. Interestingly, contrary to the reported overlapping roles of tankyrase 1 and 2 in telomerase regulation, the depletion of both enzymes worsened the persistent cohesion phenotypes observed upon knockout of either enzyme, suggesting unique and complementary roles that are not yet characterized [77].

### Tankyrases 1 and 2 in the repair of telomeric DNA

Tankyrases are not DNA-dependent enzymes. Therefore, unlike PARP1, PARP2 and PARP3, their activity is not directly triggered by DNA damage. Nonetheless, both enzymes have been implicated in the HR process, indirectly through interactions with their protein partners [69, 85, 86]. At telomeres particularly, tankyrase 1 depletion by siRNA sensitizes cells to telomeric SSBs and oxidized base damage [87]. In this study, tankyrase 1 was found to be recruited to damaged telomeric DNA through its interaction with TRF1. This interaction, mediated by the ANK domains of tankyrase 1 and the tankyrase-binding motif of TRF1 (TBM) [64], was revealed to be important for the subsequent recruitment of the SSBR and BER proteins XRCC1 and Polβ (Fig. 3B). Tankyrase inhibition using the inhibitor XAV939 impaired the recruitment of these proteins as well. However, the finding that XAV939 is also a potent inhibitor of PARP1 also suggests the involvement of PARP1 activity in DNA repair protein recruitment at telomeres [88]. Given that tankyrases do not bind DNA directly, how oxidative telomeric DNA damage is sensed remains unclear. One possibility resides in the cooperation between the DNA-dependent ART enzymes PARP1, PARP2 and PARP3. Such cooperation has already been described,  notably between PARP3 and tankyrase 1 [89, 90]. In this study, PARP3 was characterized as a positive regulator of tankyrase 1-mediated PARylation of the mitotic checkpoint protein NuMA to ensure proper spindle stabilization and telomere function. However, the relevance of this partnership following DNA damage induction, as well as a potential involvement of PARP1 and PARP2, remains to be investigated.

## DNA-dependent ARTs in the control of telomere homeostasis

Early investigations of the role of PARP1 in controlling telomere length yielded controversial conclusions. One group first observed that mouse embryonic fibroblasts (MEFs) obtained from PARP1−/− mice displayed telomere shortening as well as an increase of telomere losses and end-to-end fusions [91, 92]. Telomere shortening was not tissue-specific and was reported to be apparent in embryos and adult mice. Congruently, the pharmacological inhibition of PARP1 and PARP2 by 3AB in human HeLa cells also led to rapid telomere shortening [93]. However, a role for PARP2 in controlling telomere length was ruled out as PARP2 depletion by siRNA did not impact telomere length [93]. This confirmed prior data showing that PARP2-depleted primary mouse cells harbored normal telomere length and telomerase activity [94]. In striking contrast, another group did not observe significant telomere shortening or end-to-end fusions in PARP1-/- mouse primary cells, and telomere shortening in telomerase-deficient MEFs was not worsened by PARP1 depletion even after the fourth mouse generation [95, 96]. This discrepancy could possibly be attributed to the use of different mouse strains and the number of cell passages performed to obtain the MEFs before analysis. In fact, Samper et al. measured telomere length in true primary cells obtained after less than 2 passages and also reported an increase of end-to-end fusions when these cells were kept in culture for 26 population doublings, confirming a role of PARP1 in the long-term maintenance of telomere end-capping. Importantly, telomerase activity in PARP1−/− MEFs or 3AB-treated HeLa cells was not altered (as measured in cell extracts by TRAP assays), indicating that telomere shortening was not due to telomerase inhibition [91, 93, 95]. Yet, in contradiction with these data, an independent study using siRNA to knock down PARP1 in HeLa cells observed a decrease of telomerase activity [97]. Overall, these findings confirm a role of PARP1 and PARylation in telomere maintenance. However, whether PARP1 acts through telomerase regulation and/or via other mechanisms remains to be clarified. This is discussed in further detail below.

### PARP1 and the regulation of the telomerase enzyme

As mentioned above, whether PARP1 regulates telomerase activity is unclear and controversial. While PARP1 depletion in mice and its inhibition in human HeLa cells were reported to not impact telomerase activity, the use of PARP1 siRNA in HeLa cells did yield a decrease in activity [91, 93, 95, 97]. Interestingly, Beneke and colleagues also showed that the telomeres of cells released from 3AB inhibitor treatment were elongated back to control levels, strongly suggesting that telomerase was impacted by the 3AB inhibitor. Since all of these studies used the in vitro TRAP assay, this divergence in results is most likely due to a difference in the analysis and interpretation of the data. When assessing telomerase activity, two enzymatic parameters can be measured. The first parameter is telomerase “activity,” which indicates the total incorporation of nucleotides during the time of the experiment and is reflected by the band intensities on the TRAP gel. The second parameter is the “processivity,” which is defined as the number of telomere repeats added by the enzyme before its dissociation from the substrate. This is revealed by the number of bands on the TRAP gel [98, 99]. By measuring band density, Ghosh and colleagues observed that telomerase activity was impacted by PARP1 depletion while the other studies clearly showed that telomerase processivity was not affected. Although it remains to be confirmed and the mechanisms further investigated, these data suggest that PARP1 and its activity are involved in controlling telomere repeat addition by telomerase. Other findings in the literature hint at some hypotheses.

First, TERT was shown to harbor a functional PBM located in its C-terminal extension domain that mediates the binding of the telomerase to DNA [100–103]. Like the DNA repair protein XRCC1, the PBM of TERT could possibly mediate its recruitment to telomeric DNA during replication and ensure telomeric repeat addition. Through this mechanism, PARP1 would therefore play the same role as tankyrase 1 in the regulation of telomerase (Fig. 3A). However, the cellular conditions in which such regulation by PARP1, rather than tankyrase 1 would be necessary, remains to be uncovered.

A second mechanism involves the control of *TERT* gene expression by the Kruppel-like transcription factor 4 (KLF4), which maintains hTERT levels in human embryonic stem cells and cancer cells [104]. An interaction between the DNA-binding domain of KLF4 and the BRCT domain of PARP1 was recently reported [105]. Importantly, PARP1 knockdown leads to a decrease of hTERT expression and subsequent TERT activity while its overexpression stimulates *hTERT* transcription. Very interestingly, PARP1 activity is not required for *hTERT* transcription (Fig. 3A). Instead, H_2_O_2_-mediated PARylation disrupts PARP1/KLF4 interaction and decreases hTERT expression [105] (Fig. 3A). PARP1 is therefore proposed to promote KLF4 binding to its promoter to maintain TERT expression in stem and cancer cells, thereby contributing to pluripotency and cell proliferation.

Similarly, PARP1 knockdown in HeLa cells led to an inhibition of the telomerase-associated protein TP1/TEP1 expression [97]. TP1/TEP1 is a component of the telomerase ribonucleoprotein complex and interacts with the TR RNA component [106, 107]. However, its role in telomerase function is not known, preventing from connecting directly the impact of PARP1 on the protein expression and telomerase activity. Clearly, this aspect of PARP1-dependent telomerase regulation requires further investigation.

Finally, another mechanism worth mentioning is the regulation of telomerase assembly through the targeting of the subunits of its holoenzyme. Telomerase RNA hTR contains a 3’ H/ACA domain that is essential for the biogenesis of telomerase [108]. This domain associates with proteins DKC1, GAR1, NHP2 and NOP10 to form the H/ACA ribonucleoprotein complex. Global analyses of ADP-ribosylation protein targets revealed that two of the components of the H/ACA ribonucleoprotein complex, DKC1 and GAR1, are PARylated [109]. A recent study described the impact of DKC1 and GAR1 PARylation on their binding to telomerase RNA [110] (Fig. 3A). Surprisingly, the authors reported an increase of hTR levels in the telomerase complex upon PARP1 depletion. This underscores the complexity of the mechanisms of PARP1-mediated telomerase regulation and emphasizes the need for more studies.

### Toward a potential role for PARP3 in the regulation of the telomerase enzyme

Very few studies have investigated the roles of PARP3 in telomere maintenance and telomerase regulation. PARP3 knockdown in monkey COS-1 cells spontaneously increases the number of telomere losses and sister telomere fusions [90]. Additionally, the decrease of PARP3 levels in human lung carcinoma and osteosarcoma cells correlates with an increase of telomerase activity [111]. However, it is not yet known whether this leads to significant and stable telomere elongation with time, though it was independently shown that cancer cells harboring low telomerase activity also displayed high PARP3 expression levels [111]. Although these results seem to indicate that PARP3 could act as a negative regulator of telomerase activity, the mechanism remains to be determined.

### PARP1 in the replication of telomeric DNA

PARP1-deficient cells harbor an increased level of telomere signal-free ends [91, 92, 95]. This finding points toward the interesting possibility that the telomere shortening observed in the absence of PARP1 or its activity is directly attributed to failed telomere replication. This could explain the rapid telomere shortening rate reported upon PARP1 inhibition in HeLa cells [93], greater than that observed upon TERT depletion. Telomeres are indeed prone to replication stress and have been reported to resemble common fragile sites [12]. The single-stranded telomeric TTAGGG repeats can fold into a G4 structure. G4s are formed via the pairing of Gs by Hoogsteen bonds establishing planar tetrads. Stabilization of G4 structures using small G4 ligands triggers telomere fragility and loss [112, 113]. The RecQ helicases Werner and Bloom (WRN and BLM) are also capable of unwinding G4s at telomeres [114]. BLM deficiency increases G4 structure levels both at telomeres and throughout the genome and also slows down the replication of the telomeric lagging strand, which leads to telomere fragility [113, 115]. Similarly, depletion of WRN, which also carries an N-terminal 3′-5′ exonuclease activity, leads to an enrichment of G4s at telomeres and lack of its helicase activity triggers a loss of the 3′ single-strand G-rich overhang and telomere loss [116]. WRN and BLM interact with TRF2 and TRF1 respectively [117, 118]. However, the mechanisms of their recruitment when a replication fork encounters a G4 are not yet clear.

Interestingly, PARP1 can bind G4s in vitro, which triggers its activity [119]. Moreover, PARP1 was reported to be activated at telomeres upon G4 stabilization by the ligand RHPS4 and its knockdown led to persistent telomere dysfunction upon RHPS4 treatment, suggesting a role for PARP1 in the resolution of G4 structures [112]. Remarkably, WRN and BLM were identified in mass spectrometry analyses for the identification of proteins that bind to or are modified by PAR [19, 120]. Although no follow-up studies confirmed PARylation of BLM, WRN was demonstrated to interact with and be PARylated by PARP1 and a PBM was identified within its exonuclease domain [121, 122] (Fig. 3C). However, the impact of PARylation or PAR binding on G4 unwinding is still currently unknown. Nonetheless, the activation of PARP1 upon binding to G4 structures could trigger the recruitment of WRN to promote their unwinding, similarly to the mechanism of PARP-dependent recruitment of the DNA repair protein XRCC1 upon SSB. This remains to be tested. It is noteworthy that PAR binding to WRN’s exonuclease domain led to an inhibition of this activity, suggesting that while PARP1 may promote telomeric G4 unwinding, it may also protect telomeres from potentially harmful WRN activity. Another mechanism arises from a very recent study that demonstrates the PARylation of TRF1 by PARP1 in vitro and in vivo during S phase to promote WRN and BLM recruitment via PAR interaction [123]. Whether these mechanisms involve the direct recruitment of G4 resolvases by PARP1 or indirect recruitment through TRF1 targeting, the absence of PARP1 would consequently prevent removal of these deleterious structures, leading to replication fork collapse, telomere fragility [123] and telomere loss. This would therefore explain the telomere shortening that is observed upon PARP depletion or inhibition [91, 92, 95].

### Roles of PAR metabolism in the maintenance of telomeres via the ALT pathway

Alternative lengthening of telomeres (ALT) is a homology-directed, repair-based telomere elongation mechanism that uses HR for de novo synthesis of telomere repeats. ALT maintains telomere length in 10–15% of human cancers that are telomerase-negative [124, 125]. A recent report demonstrated the importance of PARylation metabolism in the promotion of ALT mechanisms [126]. Indeed, PARP activity inhibition by Olaparib treatment led to an increase of ALT-associated PML bodies (APBs), telomere sister chromatid exchanges (T-SCEs) and extra chromosomal telomere signals (ECTS), which are hallmarks of ALT. Accordingly, the inhibition of PARG triggered a reduction of APBs, T-SCEs and ECTS while eliciting telomere shortening. The depletion of ARH3 led to the same phenotypes, and PARG inhibition in ARH3-depleted cells further decreased APB formation [127]. More specifically, the chromatin remodeling factor HIRA was identified as a new PAR binding partner [126]. HIRA deposits histone H3.3 at ALT telomeres and was demonstrated to compensate for the lack of the factor ATRX, which is often mutated in ALT cancer cells [128, 129]. HIRA’s binding to PAR seems to be crucial for the deposition of histone H3.3, an important step during homology-directed repair in ALT cells. Interestingly, data obtained using PARP1 shRNA suggested the sole involvement of PARP1 activity in this process. However, the mass spectrometry analysis of the PAR-regulated ALT proteome also revealed the presence of PARP2. Moreover, previous work identified PARP2 at ALT telomeres specifically while no PARP2 was detected in telomerase-positive cells [94]. This could therefore suggest a role for PARP2 in the ALT pathway independent of histone deposition by HIRA. This seminal work not only highlights the importance of a balance between PAR synthesis and degradation, but also reveals an entirely new area of research to investigate the roles of PARP in ALT-dependent cancers and extend the potential use of PARP inhibitors, already used against HR-deficient tumors, in the treatment of ALT cancers particularly.

## DNA-dependent ARTs in the preservation of telomere integrity upon genotoxic stress

The shelterin complex’s primary role is to repress DDR at chromosome ends. This aspect of shelterin function has been reviewed in previous work and will therefore not be covered in this review [1, 7, 130]. Yet, like any other genomic loci, telomeric DNA can be impacted by DNA damage that can occur both endogenously and exogenously. In fact, their TTAGGG repeats predispose telomeres to replication stress and make them highly susceptible to oxidative stress [131]. Telomeres therefore necessitate the action of DNA repair proteins to preserve their integrity. To what extent the presence of shelterin on the telomeric DNA affects the repair of DNA damage occurring within telomeres remains unknown. However, considerable progress in this area has been made within the past few years, owed largely to the development of tools that target DNA damage directly to telomeres, and increasing evidence brings ART enzymes to the forefront of telomeric DNA repair [132, 133].

### PARP1-dependent alt-NHEJ for the repair of internal telomeric DSBs

DSBs occurring within the genome are mostly repaired by the c-NHEJ pathway, unless they arise during the S or G2 phases of the cell cycle during which sister chromatids are available. However, it is well documented that c-NHEJ is blocked at telomeres, mostly by TRF2. TRF2 stimulates t-loop formation and sequesters the chromosome end, preventing its recognition as a DSB and processing via c-NHEJ [1, 2, 134]. TRF2 was also found to directly prevent Ku70/Ku80 tetramerization at telomere ends [135] and its depletion leads to DNA end exposure and telomere head-to-head fusions [1, 136–138]. Accordingly, c-NHEJ was not found to repair internal DSBs generated by the endonuclease FokI in fusion to TRF1, most likely due to TRF2’s impact on Ku70/Ku80 binding activity along the telomeric repeats [133]. Whether TRF2 similarly constitutively represses PARP3 binding to the internal DSB ends to also counteract its role in assisting the Ku70/Ku80 recruitment has not been tested.

The obstruction of c-NHEJ during the repair of internal DSBs does not exclude the intervention of other mechanisms. In fact, thanks to the use of the FokI-TRF1 construct, it was clearly demonstrated that both HR and PARP1-dependent alt-EJ could repair FokI-induced DSBs [133] (Fig. 3B). Accordingly, PARP1, PARG and PAR were detected at FokI-induced DSB sites [126]. Both pathways rely on sequence homology, and telomeric DNA repeats offer the perfect substrate for  this to occur upon 5′ end resection. The repair of FokI-induced DSBs through HR was illustrated by an increase in T-SCEs and greater telomere length heterogeneity as well as the formation of ECTS, which are indicative of the ALT pathway governing telomere lengthening in telomerase-negative cancer cells.

PARP1 depletion or inhibition by the PARP inhibitor Olaparib induced telomere shortening and persistence of telomere dysfunction as well as an increase of ECTS, suggesting that PARP1 may repress telomeric internal DSB processing via HR. Additionally, PARP1 depletion had less of an impact than its inhibition. As suggested by the authors, this could be attributed to the PARP trapping mechanism induced by PARP inhibition that is more of an impediment for the cells than the protein depletion itself [133, 139]. However, it is possible that PARP2 could also fulfill part of the repair requirement. It was indeed recently reported that despite its low activity, PARP2 can ensure the recruitment of DNA repair proteins XRCC1, Ligase III and Polβ onto oxidized chromatin, thereby promoting efficient DNA repair [140]. Moreover, PARP2 is mostly activated by 5′ phosphorylated ends and could therefore directly promote end ligation via Ligase III recruitment [34]. Further studies are needed to bring evidence to these hypotheses. It is noteworthy that alt-EJ at telomeres was observed in cells expressing Ku70/Ku80, contrary to what was reported genome-wide [61–63, 133]. This highlights the singularity of telomeres and the importance of TRF2-mediated Ku70/Ku80 repression, which favors annealing-dependent head-to-tail over detrimental head-to-head telomere fusions.

### PARP1 and PARP2 in the repair of oxidative DNA damage

The abundance of Gs in the telomeric lagging strand makes it particularly sensitive to oxidative stress that can trigger progressive telomere shortening, telomere uncapping and eventually genomic instability [131, 141–143]. Given the crucial roles of PARP1 and PARP2 in the repair of oxidized DNA bases and single-strand breaks via BER and SSBR, it is most likely that both enzymes function at telomeres subjected to oxidative stress. However, the data available in the literature thus far are not extensive and therefore several questions persist. One of the earliest studies in this area reported that PARP1 depletion in MEFs exposed for 48 h to sublethal doses of Arsenite-induced reactive oxygen species (ROS) enhanced telomere attrition and telomere loss when compared to PARP1-expressing cells [144] (Fig. 3B). In line with this data, lack of PARP1 led to an augmentation of telomere loss and end-to-end fusions following X-ray exposure of MEFs and HeLa cells, while knockdown of PARG protected telomeres from telomeric aberrations following gamma irradiation [45, 145]. Importantly, PARP1 and TRF2 directly interact via the BRCT domain of PARP1 and the Myb domain of TRF2 in vitro, and PARP1 was recruited to telomeres upon H_2_O_2_ and X-ray treatments, partially dependent on its interaction with TRF2 (Figs. 2 and 3B). Interestingly, PARP1 was able to PARylate the TRFH domain of TRF2, impacting TRF2 DNA binding in vitro [145]*.* However, the impact of TRF2 PARylation upon oxidative damage in cells was not investigated. PARP2 was only slightly enriched at telomeres following H_2_O_2_ treatment. Yet, similarly to PARP1, PARP2 was found to interact with TRF2 via the Myb domain and to PARylate its TRFH domain in vitro, impacting its DNA binding activity. Here again, the effects of TRF2 PARylation by PARP2 were not reported in cells [94]. Finally, a comprehensive profiling of PARylation sites using mass spectrometry identified four serines (one being present in the Myb domain) and one histidine (in the TRFH domain) as PARylation targets [146]. However, these sites were not confirmed experimentally. Nonetheless, these studies clearly demonstrate the involvement of PARP1 and PARP2 in the repair of oxidative lesions at telomeres, but further investigations are required. The recent development of a tool that specifically induces 8-oxoguanine (8-oxoG) lesions at telomeres will certainly help in this endeavor [147]. This system, called the Fluorogen-Activated Peptide (FAP) tool,  utilizes the stable expression of the FAP peptide in fusion with TRF1 for its convenient targeting to telomeres. The FAP peptide binds the malachite green 2i dye (MG2i), which generates singlet oxygen radicals (^1^O_2_) upon exposure to a 660 nm wavelength light. This ^1^O_2_ subsequently leads to 8-oxoG lesion formation specifically at telomeres [132]. Using the FAP system, PARP1 was found to be activated locally at telomeres and the DNA repair protein XRCC1 efficiently recruited [132]. This tool offers an opportunity to address remaining questions regarding the individual roles of PARP1 and PARP2 in the repair of oxidized telomeric DNA, their telomeric targets and the subsequent impact on their binding activities.

### PARP1 functions during replication stress at telomeres

Telomeres are difficult-to-replicate loci and are prone to replication stress. Replication stress at telomeres promotes telomere fragility that appears as multi-telomeric fluorescent signals on metaphase spreads [12]. The problems encountered by the replication fork when replicating telomeres are inherent from their structure and DNA sequence. When telomeric DNA is single-stranded, for example during replication, the G-rich strand can form stable G4 structures that present major barriers for forks. Another obstacle is the telomeric t-loop that is initially formed to protect the DNA ends from the DDR machinery but must be transiently dismantled during S-phase to avoid fork stalling. G4 and t-loop resolution are ensured by the helicases RTEL1, BLM and WRN [12, 113, 148]. During S-phase, RTEL1 is recruited via binding to the TRFH domain of TRF2. Depleting RTEL1 or preventing its binding to TRF2 leads to aberrant t-loop excision by the SLX4 nuclease and telomere loss as telomeric circles (TCs) [113, 148]. Remarkably, in this context, PARP1 activity is rather detrimental. Indeed, t-loop persistence triggers replication fork reversal [149] which is promoted by PARP1 activity [150, 151]. Inhibiting or depleting PARP1 in RTEL1-deficient cells prevented fork reversal and subsequent telomere defects. During fork reversal, PARylated PARP1 interacts with RECQL1 helicase, whose activity is necessary for fork restart (Fig. 3D). Therefore, preventing PARG from degrading PAR promotes telomere defects if RTEL1 is depleted and the t-loop unresolved [149]. This work provides an example of the importance of the careful balance of PAR synthesis and degradation and also provides an attractive therapeutic option for RTEL1-mediated telomeropathies, such as Hoyerall–Hreidarsson syndrome (HHS) and Dyskeratosis congenita (DKC) [152–156]. It is noteworthy that the 5′ double-to-single DNA transition at the base of the t-loop could be a substrate for PARP1 [34]. Additionally, the recruitment of the nuclease SLX4, responsible for the t-loop cleavage in RTEL1-depleted cells, is partly dependent on PARP1 activity [157]. The rescue of telomere dysfunction following PARP1 inhibition observed by Margalef et al. could therefore also be attributed to a decrease of SLX4 recruitment directly to the t-loop (Fig. 3D).

Finally, PARP1 has also recently been implicated in the mechanisms of DNA repair following unresolved, G4-mediated replication stress [158]. In this case, the persistence of the G4 observed following the depletion of the BLM helicase was responsible for the formation of unreplicated DNA gaps processed by SLX4. The subsequent DSBs were therefore demonstrated to be partly repaired through PARP1-dependent alt-NHEJ [158] (Fig. 3D).

## Concluding remarks

In this review, we shed light on the ever-expanding functions played by five members of the ART family particularly at telomeres. While the functions of tankyrase 1 and 2 roles in the maintenance of telomeres via telomerase regulation and telomere cohesion resolution are well understood, their potential roles in assisting in the repair of DNA damage at this specific locus is still unclear. Moreover, it is becoming clear that the unique structure and protein content of telomeres have an impact on the canonical DNA repair function of the DNA-dependent ART enzymes while promoting novel roles, notably telomerase-dependent or -independent telomere length regulation. Additionally, the recent findings of the factors HPF1 and ARH3 and their crucial roles in the repair of DNA lesions by PARP1 and PARP2 may give rise to novel telomere-specific functions. Telomere dysfunctions are central to many human diseases, including cancer, aging-related illnesses and telomeropathies. The proteins involved in telomere maintenance therefore are promising therapeutic targets in the treatment of these disorders. Telomerase inhibitors are currently investigated while PARP inhibitors are already widely used for cancer therapies. Understanding the roles of ART enzymes in telomere biology not only expands the opportunities for the use of established PARP inhibitor molecules in other diseases but also allows for the potential development of combination therapies that could mitigate PARP inhibitor resistance.

## Data Availability

Enquiries about data availability should be directed to the authors.
